# A Retrospective Study on Traumatic Elbow Dislocation in Adults in a Tertiary Hospital in Riyadh, Saudi Arabia

**DOI:** 10.7759/cureus.37554

**Published:** 2023-04-14

**Authors:** Abdulaziz F Alkheraiji, Ismail Almogbil, Moath Aljohani, Abdulmalik B Albaker, Hussain Algawahmed

**Affiliations:** 1 Department of Orthopaedics, Majmaah University, Majmaah, SAU; 2 Department of Family and Community Medicine, College of Medicine and Medical Sciences, Qassim University, Al Qassim, SAU; 3 Department of Orthopaedics, College of Medicine, Majmaah University, Majmaah, SAU; 4 Hand, Elbow & Shoulder Surgery, Johns Hopkins Aramco Healthcare, Dhahran, SAU

**Keywords:** computed tomography, incidence, retrospective review, radial head injuries, elbow dislocation

## Abstract

Background

This retrospective chart review aimed to ascertain the frequency and characteristics of radial head fractures in adults who presented to our emergency department with elbow dislocation.

Methodology

This study was conducted in a single tertiary trauma center in Riyadh, Saudi Arabia, between July 2015 and July 2020 to identify traumatic elbow dislocation in adults. Patients were identified after thoroughly examining the hospital’s electronic X-ray database. In addition, computed tomography (CT) was used to assess complete ulnohumeral joint dislocation. In total, 80 patients between the ages of 18 and 65 were evaluated for a radial head fracture. Various variables were examined.

Results

Of the 80 patients included, the mean age with standard deviation was 36.9 ± 8.8 years, and all patients were males. Nearly all patients with elbow dislocation had some form of posterior dislocation, including posterolateral (81.3%), posterior (10%), and posteromedial (7.5%) dislocation. The radial head fracture was identified in 48 (60%) cases. Radiographs were sufficient to diagnose 91.3% of radial head fractures, while the remaining 8.8% required additional CT.

Conclusions

Based on X-ray or CT findings, radial head fractures were found in more than half of traumatic elbow dislocations. In addition, most cases were diagnosed as an elbow dislocation and radial head fracture using only plain radiography, while some required additional CT. Based on these findings, we recommend routine CT to detect suspected elbow dislocation and avoid missing subtle injuries.

## Introduction

In adults, elbow dislocation is the second most common form of dislocation, followed by glenohumeral joint dislocation [1]. Elbow dislocations are classified as either simple or complex depending on their association with fractures. Complex dislocations are associated with fractures of the distal humerus, radial head, ulna, or coronoid process. Posterolateral and posterior dislocations account for approximately 90% of all elbow dislocations, whereas lateral, medial, and anterior dislocations are less common injuries [1]. Furthermore, radial head fractures are found in approximately 20% of elbow traumas. They commonly occur after sustaining a fall on an outstretched hand with wrist extension and forearm pronation [2].

The presence of radial head fractures can change the management approach for elbow dislocations. Elbow dislocations associated with coronoid fracture alone with less than 50% height involvement can be treated conservatively in the majority of cases with a good outcome [3]. Conversely, the association of coronoid with radial head fracture almost always requires surgical intervention to restore the stability of the elbow [4]. However, surgical interventions increase postoperative complication rates, including elbow stiffness, infection, heterotopic ossification, ulnar nerve neuropathies, and recurrent elbow instability [5,6].

Despite the high trauma rate, there is a tendency to overlook elbow-associated injuries in the Saudi orthopedic community. To our knowledge, no previous studies have investigated the incidence or prevalence of radial head fractures in elbow dislocation in Saudi Arabia. This study aimed to examine the incidence and characteristics of radial head fractures in adults who presented to our emergency department with elbow dislocation. We hypothesized that the characteristics of Saudi Arabians with elbow dislocations associated with radial head fractures would differ from those in the previously published literature. The findings from this study might assist in providing a better understanding of elbow dislocation and associated fractures in the Saudi Arabian population. Additionally, data collected during this study will be added to the database on elbow dislocations in Saudi Arabia, which is lacking such information due to the rarity of this information being documented. Therefore, ultimately, these findings might help policymakers in drafting specific legislation and guidelines which could improve patient outcomes.

## Materials and methods

This retrospective chart review was conducted in a single tertiary trauma center to identify traumatic elbow dislocation in adults between July 2015 and July 2020 in Riyadh, Saudi Arabia. This period covers the period during which a fellowship-trained upper extremity subspecialist joined the center. Ethical approval to report this case/these cases was obtained from the Ministry of Health of Saudi Arabia (IRB approval number: 21-68 E). Therefore, all available data were collected during this period. The study population included patients who presented to this tertiary hospital in Riyadh with acute elbow dislocation.

Patients were identified via a detailed review of our hospital’s electronic X-ray database. Overall, 3,301 elbow X-rays were identified and reviewed at our institute. Three experienced orthopedic surgeons evaluated X-rays. Complete ulnohumeral joint dislocation was further assessed using computed tomography (CT). Adult patients aged 18-65 were included if they had a confirmed diagnosis of acute traumatic elbow dislocation. Orthopedic surgeons widely use the modified Mason classification [6]. Using this approach, all dislocations with a radial head association, as in this study, are classified as type IV. Owing to this limitation, we used the original Mason classifications, namely, types I, II, and III [7].

Patients aged <18 years were excluded due to the tendency of these injuries to be bony rather than ligamentous. Furthermore, pediatric patients with congenital dislocations were excluded, as these patients have substantially different treatments. Patients aged >65 years were excluded because of the higher incidence of poor bone quality, low-energy trauma, and lower activity levels, all of which alter treatment preferences. Patients diagnosed with isolated radial head fractures without evidence of elbow dislocation on CT scans were also excluded. In total, 80 patients were included and evaluated in this study. The included patient files were retrieved from the medical record archives and then reviewed.

Statistical analysis

Data were analyzed using SPSS statistics for Windows version 22 (IBM Corp., Armonk, NY, USA). Categorical variables, including sex, occupation, mode of injury, dislocation side and direction, Mason classification of the radial head fracture, diagnostic modalities, and surgical procedures, are presented as frequencies and proportions (n, %). Continuous variables (age) are presented as means and standard deviations (mean ± SD). Falls from different heights were grouped into one variable, and the Mason classification for radial head fracture was graded as follows: I, simple nondisplaced; II, simple displaced; and III, comminuted fracture. Fisher’s exact tests assessed the association between the initial diagnostic modalities and whether the case was managed conservatively or surgically. Pearson’s coefficient (p-values) <0.05 were considered significant.

## Results

The included patients’ mean (SD) age was 36.9 ± 8.8 years, and all patients were males. The majority of the patients (78.3%) worked as laborers or undertook other forms of manual labor, including holding jobs as carpenters, electric technicians, and plumbers. Another 13.3% of patients were involved in some form of office work, including holding jobs such as accountants, company employees, or students. The most commonly reported injury method was falling from a height (88.8%), followed by road traffic accidents (RTAs, 8.8%). Injuries associated with crushing accounted for 2.6% of injuries (Table 1).

**Table 1:** Demographic characteristics and mode of injury among the cases of elbow dislocation (n = 80). Data are presented as frequencies and proportions or the mean ± SD. N: frequency; SD: standard deviation; RTA: road traffic accident

Variable		N (%), 80 (100)
Age (years)		36.9 ± 8.8
Sex	Male	80 (100.0)
	Female	0 (0.0)
Occupation	Laborers	47 (78.3)
	Office workers	8 (13.3)
	Drivers	5 (8.3)
Mode of injury	Fall from height	71 (88.8)
	Crushing injury	7 (8.8)
	RTA	2 (2.6)

Nearly all included patients had some form of posterior dislocation, including posterolateral (81.3%), posterior (10%), and posteromedial (7.5%) dislocation. Two-thirds of the cases had dislocation of the left side (Table 2).

**Table 2:** Characteristics of elbow dislocation and radial head fracture (n = 80). Mason classification: I, simple nondisplaced; II, simple displaced; III, comminuted fracture. N: frequency

Variable		N (%), 80 (100)
Dislocation side	Right	54 (67.5)
	Left	26 (32.5)
Dislocation direction	Posteromedial	65 (81.3)
	Posterior	8 (10.0)
	Posterolateral	6 (7.5)
	Anterior	1 (1.3)
Presence of a radial head fracture		48 (60.0)
Mason classification of radial head fracture	I	6 (12.5)
	II	13 (27.1)
	III	29 (60.4)
Presence of a posterior capitellar defect		16 (23.9)

In all traumatic elbow dislocations, a radial head fracture was identified in 48 (60%) of the cases based on radiological X-ray or CT findings. Among them, 27.1% and 60.4% had Mason classifications of grade II or III, respectively. The majority of radial head fracture cases (91.3%) were initially diagnosed based on plain X-rays. The remaining 8.8% required additional CT scans. Posterior capitellar wear along with dislocation (Osborne-Cotterill lesion) was observed in 24% of the cases (Table 3).

**Table 3:** Initial diagnostic modalities and management offered for cases of elbow dislocation (n = 80). n: number

Diagnostic modality		Management		Total	P-value
		Conservative	Surgical		
X-ray	N (%)	25 (30.3%)	48 (60.5%)	73 (91.3%)	0.685
CT	N (%)	3 (3.9%)	4 (5.3%)	7 (8.8%)	

Two-thirds (66%) of the cases were managed by surgical intervention. Management was not dependent on the modality used for the initial detection of the dislocation, whether it was X-ray or CT (p < 0.05). Most patients underwent radial head replacement or fixation in conjunction with other frequent procedures, including lateral ulnar collateral ligament (LUCL) repair. Coronoid process fixation was performed using screw fixation or transosseous sutures in 9.6% of the cases. Medial collateral ligament (MCL) repair was only required in 1.9% of the cases (Table 4).

**Table 4:** Types of surgical interventions applied in patients with elbow dislocation who received a surgical intervention (n = 52). *: Multiple procedures were conducted for the same case. N: frequency; LUCL: lateral ulnar collateral ligament; RH: radial head; ORIF: open reduction and internal fixation; MCL: medial collateral ligament

Surgical procedure*	N (%), 52 (100)
RH replacement	35 (67.3)
LUCL repair	22 (44.0)
Coronoid process ORIF	5 (9.6)
RH ORIF	3 (5.7)
MCL repair	1 (1.9)
Closed reduction and external fixation	1 (1.9)

## Discussion

Radial head fractures may be associated with elbow dislocation. The incidence of radial head fractures is 2.5-2.9 per 10,000 people per year, i.e., present in 33% of all elbow fracture cases. In addition, it is a high-energy trauma. It can present with partial, displaced, complete, or comminuted fractures that may lead to loss of bony support and subsequent instability of the elbow joint [8,9].

In Saudi Arabia, there are no officially published data on the incidence of elbow dislocations or radial head fractures; however, a study on the epidemiology of fractures and dislocations among urban communities of the eastern province was conducted in Dammam, Saudi Arabia. Sadat-Ali et al. found that RTA was the primary cause of general trauma cases. The study found that the number of elbow injury cases was relatively high in the community, mainly due to its increasing birth rates and increased number of RTAs. Upper extremity fractures occurred in 555 cases, and only 4% sustained elbow dislocation [10].

In this study, we found that 88% of dislocations were due to falls from a height, but we did not record whether any were due to sports injuries. RTAs accounted for only 8.8% of the cases. In the United States, the estimated incidence of elbow dislocations is 5.21 per 100,000 person-years, according to the national database, with sports injuries accounting for nearly half of the cases [11]. Robinson et al. reported that a fall from a height was a significant cause of dislocation, followed by sports injuries [1].

In our study, 100% of elbow dislocations occurred among men. In the study by Sadat-Ali et al., 14 male and nine female patients had elbow dislocation [10]. Robison et al. found that the number of male patients with elbow dislocation was double that of female patients [1]. According to another study, the male-to-female ratio was 1.9:1.13. These numbers vary between male and female patients mostly because elbow dislocation is a high-energy trauma associated with heavy-duty work, which is a male-dominant job. In Saudi Arabia, female participation in competitive sports and heavy labor is rare [12].

Mühlenfeld et al. reported that the average age of patients with elbow dislocations was 48.5 (range = 18-86) [13]. In the present study, the average age was 36.9 years. This younger age might be because the population included in this study were mainly patients who sustained occupational injuries. However, age did not dictate surgical treatment or affect surgical management [13].

In our study, radial head fractures among traumatic elbow dislocation patients accounted for 48 (60%) cases. Most cases (91.3%) were diagnosed as an elbow dislocation and radial head fracture using plain radiography alone, and 8.8% of the patients required additional CT for diagnosis. Based on these findings, we recommend routine CT to detect suspected elbow dislocation and avoid missing subtle injuries such as posterior capitellar wear (Osborne-Cotterill lesion) and radial head fracture. Posterior capitellar wear and dislocation were observed in 24% of the cases. A depressed fracture involving the posterolateral humeral condyle indicates this lesion [14]. Missing such lesions can have a long-standing effect on the patient, such as posterolateral rotatory instability of the elbow [15]. CT is also necessary for assisting in perioperative planning [5].

Robinson et al. found that posterolateral and posterior dislocations account for approximately 90% of all elbow dislocations, while lateral, medial, and anterior dislocations are less common injuries [1]. Nearly all included patients in our study had some form of posterior dislocation, including posterolateral (81.3%), posterior (10%), and posteromedial (7.5%) dislocation. These findings are similar to those of recently published data [2].

Two-thirds (66%) of the patients were managed using surgical interventions. Most patients underwent radial head replacement (70%), while fixation was performed in 12% of the cases. Other frequently performed procedures, including LUCL repair and coronoid process fixation, were performed as required. MCL repair was less often needed.

This study has some limitations worth mentioning. First, the single-center design is subject to selection bias; however, because the included patients were mostly manual laborers, it might represent the population who commonly experience such injuries. Moreover, our study lacked potentially essential data such as the time between surgery and original trauma, patients’ hand dominance, and final range of motion. However, because the main objective of this study was to describe trends in elbow dislocation and associated radial head injuries, such additional information did not affect our outcomes. Finally, in accordance with the findings of this study, we highly recommend routine CT to detect suspected elbow dislocation to avoid missing subtle injuries.

## Conclusions

Based on X-ray or CT findings, radial head fractures were discovered in more than half of acute elbow dislocations. Most patients were identified by plain radiography alone as elbow dislocation and radial head fractures, while several diagnoses required further CT. Based on these findings, we advise routine CT to identify possible elbow dislocations and prevent minor injuries from going unnoticed.
