# Correction: A Retrospective Study on Traumatic Elbow Dislocation in Adults in a Tertiary Hospital in Riyadh, Saudi Arabia

**DOI:** 10.7759/cureus.c117

**Published:** 2023-05-17

**Authors:** Abdulaziz F Alkheraiji, Ismail Almogbil, Moath Aljohani, Abdulmalik B Albaker, Hussain Algawahmed

**Affiliations:** 1 Department of Orthopaedics, Majmaah University, Majmaah, SAU; 2 Department of Family and Community Medicine, College of Medicine and Medical Sciences, Qassim University, Al Qassim, SAU; 3 Department of Orthopaedics, College of Medicine, Majmaah University, Majmaah, SAU; 4 Hand, Elbow & Shoulder Surgery, Johns Hopkins Aramco Healthcare, Dhahran, SAU

This article has been corrected at the request of the authors to edit data that had been mislabeled and transposed in the initial published version:

Table 1: ‘RTA’ has been replaced with ‘Fall from height’ and ‘Fall from height’ has been replaced with ‘RTA’. Table 2: ‘Anterior’ has been replaced with ‘Posterior’ and ‘Posterior’ has been replaced with ‘Anterior’. Table 2 legend: ‘60.4% and 27.1%’ has been replaced with ‘27.1% and 60.4%’. Table 4 title: ‘75’ has been changed to ‘52’. Table 4: LUCL repair has been changed from ‘10 (19.2)’ to ‘22 (44.0)’.

The authors deeply regret that these errors were not identified and addressed prior to publication.

 

